# Development and Implementation of an Integrated Model of Perinatal Diabetes Education and Management to Improve Maternity Outcomes and Health Equity

**DOI:** 10.1089/heq.2023.0196

**Published:** 2024-02-02

**Authors:** Melanie Browning, Shahrin Sharikha, Kristopher Wu, Stacee Silagi, Victoria Greenberg, Loral Patchen

**Affiliations:** ^1^ Women's and Infants' Services, MedStar Washington Hospital Center, Washington, District of Columbia, USA.; ^2^ Georgetown University School of Medicine, Washington, District of Columbia, USA; ^3^ Healthcare Delivery Research, MedStar Health Research Institute, Hyattsville, Maryland, USA.

**Keywords:** diabetes, pregnancy, intervention, integrated care models

## Abstract

Diabetes mellitus (DM) confers unique risks during the perinatal period, contributing to maternal, fetal, and neonatal morbidity and mortality. Integrating DM education and management services with obstetrical care offers key advantages for birthing individuals. The purpose of this study is to describe the development and implementation of a perinatal DM program at a large ambulatory practice serving a diverse population. Understanding this approach and program workflow may facilitate adoption of similar services in other care settings.

## Introduction

### Role of perinatal diabetes in maternal health inequity

Diabetes mellitus (DM), a metabolic disease of carbohydrate intolerance, commonly jeopardizes maternal, fetal, and neonatal health during the prenatal period, postpartum period, and beyond (Table 1).^1–11^ In the United States, the rate of gestational diabetes mellitus (GDM) increased by 30% from 2016 to 2020^6^ (7.8 per 100 births) and that of pre-GDM by 27% from 2016 to 2021 (10.9 per 1000 births); the majority of pre-GDM were type 2 diabetes mellitus (T2DM).^7^ Pregnant individuals with type 1 diabetes mellitus (T1DM) have a mortality rate 2–3 times higher than that of nonpregnant individuals with T1DM and 5–20 times higher than that of the overall pregnant population.^12^

**Table 1.** Maternal and Fetal/Neonatal Risks Associated with Gestational Diabetes Mellitus and Diabetes Mellitus

	Gestational diabetes mellitus^4,8,9,11^	Diabetes mellitus^5,6,10,11^
Definition	First diagnosed in the latter trimesters of pregnancy. Generally resolves after delivery, but can have long-term adverse effects	First diagnosed before pregnancy and is divided into two groups. T1DM results from autoimmune destruction of pancreatic β cells and is characterized by early onset with necessary insulin therapy. The more common T2DM is associated with peripheral insulin resistance, insulin deficiency, and obesity and is characterized by late onset. Risks are reportedly more common in pregestational DM than in gestational DM
Maternal risks	*Short-term risks include* gestational hypertension, spontaneous abortion, preterm labor, pre-eclampsia, shoulder dystocia, polyhydramnios, cesarean delivery	
	*Long-term risks include* T2DM, metabolic syndrome, DM-related end-organ damage (cardiovascular, kidney, liver, and retinal disease)	
Fetal and neonatal risks	*Short-term risks include* prematurity, neonatal intensive care unit admission, macrosomia, hypoglycemia, hyperbilirubinemia, respiratory difficulties, congenital malformations, fetal demise, and neonatal death	
	*Long-term risks include* hyperglycemia, DM, impaired glucose tolerance, overweight/obesity, metabolic syndrome, and cardiovascular disease	

DM, diabetes mellitus; T1DM, type 1 diabetes mellitus; T2DM, type 2 diabetes mellitus.

In 2021, White birthing individuals had the lowest pre-GDM rate when compared with other groups.^7^ Likewise, Black and Hispanic individuals are more likely to develop T2DM than White individuals after GDM.^13^ Most neonatal outcomes are similarly disproportionate.^13^ These disparities are attributable to factors such as social drivers of health, comorbidities and chronic conditions, and access to health care and DM management programs.^13^

Disparities in perinatal DM occur in the context of staggering inequity in maternal outcomes, an urgent public health crisis of significant magnitude in the United States. In 2021, the U.S. maternal mortality rate for non-Hispanic Black (Black) birthing individuals was 2.6 times that of non-Hispanic White (White) birthing individuals, totaling 69.9 deaths per 100,000 live births.^14^ In 2020, U.S. infant mortality rates exhibited comparable trends [Black (10.38), non-Hispanic American Indian/Native American (7.68), non-Hispanic Native Hawaiian or Other Pacific Islander (7.17), Hispanic (4.69), White (4.40), and non-Hispanic Asian (3.14)].^15^

This stark contrast in maternal and infant mortality among racial and ethnic groups has persisted for several years.^14,16^ Integrated pregnancy DM care programs may offer a strategy that contributes to efforts to address this deep inequity in maternal outcomes.

Integrated pregnancy DM care programs have been shown to improve perinatal outcomes. Multidisciplinary approaches with a team that includes health care providers, educators, nutritionists, social workers, behavioral health specialists, and nurses promote patient education and self-empowerment as well as assessment for and assistance with barriers to care.^17,18^ The approach may also help mitigate the systemic inequities in maternal outcomes.

### Purpose

The purpose of this study is to describe development of an integrated perinatal DM education and management program (IP-DEMP) that is based on DM self-management education and support, or DSMES, which entails assessment and education focusing on seven self-care behaviors (healthy eating, being active, taking medication, monitoring, reducing risks, healthy coping, and problem solving).^19^ This study also shares process evaluation findings.

The IP-DEMP is encompassed under the *Safe Babies Safe Moms Initiative*,^20^ a multidisciplinary health system initiative dedicated to improving maternal and child health outcomes and health equity in the District of Columbia. This report may guide other organizations to adopt this kind of service. The institutional review board of MedStar Health provided approval for all research activity related to IP-DEMP.

## Integrated Perinatal DM Education and Management Program

### IP-DEMP implementation

**FIG. 1.**
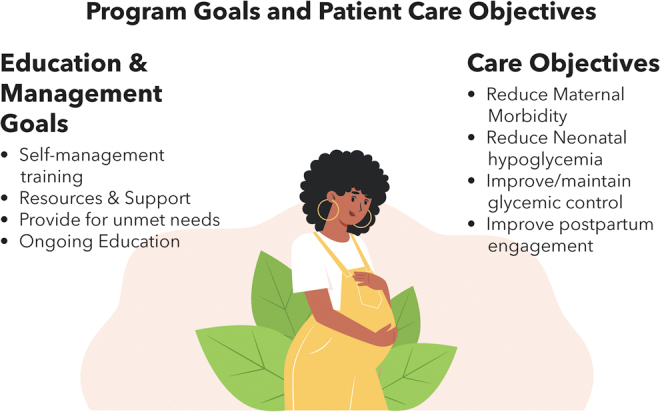
IP-DEMP goals include education and clinical outcomes. IP-DEMP, integrated perinatal diabetes education and management program.

A large academic urban hospital in the District of Columbia established an IP-DEMP to improve quality of care and patient outcomes (Fig. 1). The motivation to create the program is multifold. Before the start of the IP-DEMP, there were extended gaps between patient referral and initial DM education appointment at the endocrinology office, several weeks between follow-up appointments, no medication adjustments between prenatal appointments, and no streamlined process of coordination of care between DM educators and prenatal providers.

As such, process objectives of the IP-DEMP include reducing time between prenatal referral and first appointment with a DM educator, increasing communication between prenatal providers and educators about care plans and recommendations, and facilitating follow-up during the postpartum period. Finally, most patients with DM identified as members of historically marginalized groups and reported unmet social needs with health-harming effects. The hospital serves a large population of women who are low income and who often experience significant barriers to receiving adequate prenatal care due to social drivers of health.

The health care team felt that an integrated program would allow for care that not only provided timely coordinated DM care but also a higher level of individualized care that was responsive to social drivers of health. Overall, care for patients with DM was fragmented across the perinatal continuum and among specialty providers. IP-DEMP was identified as a promising solution to this overarching challenge.

**FIG. 2.**
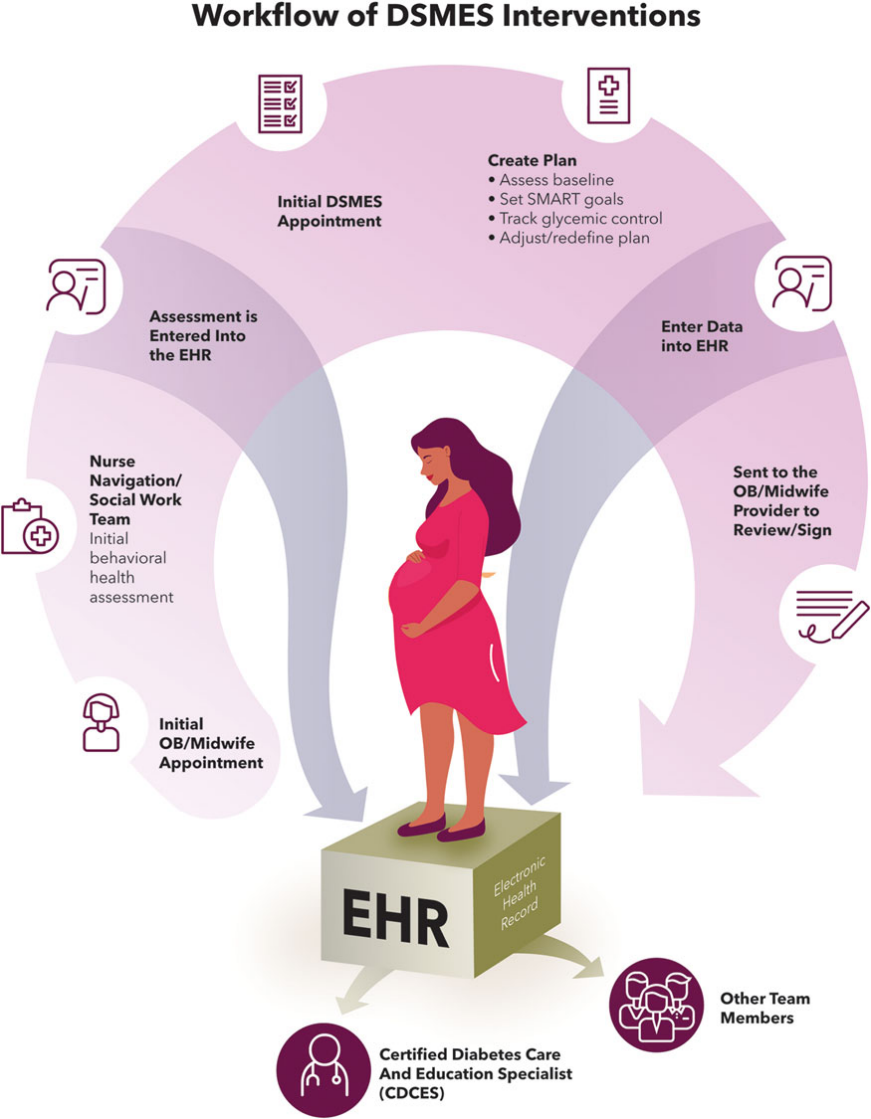
IP-DEMP workflow connects multiple team members and reflects significant integration. EHR, electronic health record; DSMES, diabetes self-management education and support.

A collaborative stepwise approach drives the program workflow (Fig. 2). The IP-DEMP team consists of two certified diabetes care and education specialists (CDCESs); a maternal–fetal medicine (MFM) physician specialist meets monthly with the team to discuss specific management plans for patients and provide ongoing clinical support. The IP-DEMP team conducts office visits for prenatal and postpartum visits. All locations are part of the same health system, including one on-site location with 2 days of in-person appointments and 3 days of telehealth appointments, and four off-site locations with solely telehealth visits.

All services are colocated, meaning they are provided in collaboration from the same obstetric and gynecological office setting. Program referrals from affiliated prenatal offices are placed through an order set in the electronic medical record (EMR), which also contains DM testing supplies organized by insurance.

### Participant engagement

Participants served in 2022 had diverse characteristics and represented local demographics. Most participants served were Black (62.5%) and had public insurance (58.2%). Many age groups were represented and 32.8% of participants were primiparous (Table 2). The referral specifies whether the patient has pre-existing DM or GDM—diagnosis follows the American Diabetes Association (ADA) and American College of Obstetrics and Gynecology diagnostic guidelines.^1,2,21^

**Table 2.** Descriptive Characteristics of Integrated Perinatal Diabetes Education and Management Program Participants

Variable	(***n***/%)	
Age, years (mean, range)	33.3	19–46
18–24	15	6.5
25–34	118	50.9
≥35	99	42.7
Race (*n*/%)		
Black	145	62.5
Asian	12	5.2
White	23	9.9
Declined to answer/unknown	15	6.5
Other race^a^	37	15.9
Ethnicity (*n*/%)		
Hispanic/Latin American^b^	14	6.0
Non-Hispanic/Latin American	173	74.6
Declined to answer/unknown	45	19.4
Insurance status (*n*/%)		
Medicaid and Medicaid HMO plans	135	58.7
Commercial^c^	95	41.3
Parity (*n*/%)		
0	76	32.8
1	52	22.4
2	55	23.7
>2	49	21.1
DM classification		
GDM	165	71.1
T1DM	14	6.0
T2DM	53	22.8
DM treatment		
Diet	92	39.8
Insulin, insulin pump	97	42.0
Insulin and medication	19	8.2
Metformin, meformin and glyburide	23	1.0

^a^ American Indian/Pacific Islander; Other.

^b^ Central American; Dominican; Hispanic; Latin American; Mexican; Puerto Rican; Spaniard.

^c^ Commercial; private.

GDM, gestational diabetes mellitus; HMO, health maintenance organization.

The IP-DEMP team member assigned to the referral directly contacts the patients to schedule their intake and provide educational materials through email. In 2022, >90% of patients were scheduled within 3 days of receiving the referral. The team attributes timely direct outreach from the IP-DEMP team as an important factor contributing to high levels of engagement. On average, 85% (range 76.6–91.6%) of participants attended their telehealth appointments with their DM educator in 2022.

IP-DEMP team members directly communicate with prenatal providers through the EMR and during perinatal office appointments, meeting jointly with patients and obstetrical providers as needed. At the initial visit, the CDCES addresses any patient concerns regarding DM management. The assessment includes age, gestational age, pregnancy history, personal/family history, pregravid weight/body mass index, current weight, gestational weight gain, review of relevant laboratories/medications/supplements, fetal growth assessments, blood glucose/food log, diet recall, and physical activity regimen.

After the assessment, the educator provides information on development of GDM, risks associated with hyperglycemia during pregnancy, how to monitor blood glucose, blood glucose targets, meal planning and consistent carbohydrate diet education, insulin use during pregnancy, and effect of physical activity on glycemic control. Using shared decision making, the educator and patient agree on dietary and lifestyle adjustments, and treatment with medication if appropriate. When needed, participants receive education on insulin preparation, injection technique, site rotation, needle disposal, insulin storage, and hypoglycemia awareness and treatment.

Before delivery, the IP-DEMP team member orients participants to postpartum recommendations and the importance of postpartum testing (for those with GDM). After delivery, the CDCES enters an order for a postpartum 2-h glucose tolerance test into the EMR and notifies the office registration team and nurse navigation team of the need for a morning postpartum appointment given that the test is completed in a fasting state. The nurse navigator calls the patient within 3 days after delivery and reminds the patients of their postpartum glucose tolerance test, which is completed in conjunction with their 6-week postpartum appointment.

Birthing individuals with pre-existing DM have a planned postpartum medication/insulin regimen. Referrals to endocrinology or primary care are coordinated 3 months before their estimated due date for continued glycemic management postpartum. Monthly engagement in program services is high and consistent among eligible patients (Table 3).

**Table 3.** Integrated Perinatal Diabetes Education and Management Process Metrics 2022

	January	February	March	April	May	June	July	August	September	October	November	December	Total
Delivery of services to referred patients													
Engaged	34	38	44	52	44	46	42	44	34	35	40	41	494
Eligible	35	39	53	55	51	47	47	50	40	37	44	47	545
Percent engaged	97.1	97.4	83.0	94.5	86.3	97.9	89.4	88.0	85.0	94.6	90.9	87.2	90.6
Prenatal appointments													
Attended	42	48	62	67	58	58	56	54	44	49	55	66	659
Scheduled	50	55	67	78	64	67	62	64	53	64	71	77	777
Percent attended	84	87	91.6	85.9	90.6	86.6	90.3	84.4	83	76.6	77.5	85.7	85.3
Postpartum appointments													
Completed	9	6	9	4	14	8	8	15	9	9	10	14	115
OGTT/endocrine indicated	11	8	15	11	20	11	12	21	10	10	13	17	159
Percent completed	81.8	75	60	36.3	70	72.7	66.7	71.4	90	90	76.9	82.4	72.3

OGTT, oral glucose tolerance test.

### Social drivers of health

Visits include ongoing discussion of barriers to care, such as lack of transportation or food and housing insecurity, and emotional wellness. Participants receive comprehensive assessments for social drivers of health and mental health needs, twice during pregnancy and again postpartum. Questions about social and environmental variables are based upon the draw from validated instruments from the EveryONE project, and emotional well-being is assessed, in part, by the Edinburgh Postnatal Depression Scale.^22,23^

If needs or barriers are identified, the DM educator assists by enrolling patients in food assistance programs through their insurance, the Special Supplemental Nutrition Program for Women, Infants, and Children (WIC), and other community resources as well as by coordinating with the referral specialist (arranges transportation through patient's insurance), social work team (addresses social needs, performs maternal mental health assessments, and provides therapy), nurse navigators (provide clinical care coordination and related patient education), and, if needed, the medical–legal partnership (addresses health-harming legal needs).

### Key IP-DEMP strengths

The IP-DEMP model streamlines early care initiation and appointment adherence through dedicated colocated services with timely direct patient outreach. Through comprehensive assessments, the IP-DEMP team identifies participants' knowledge gaps and individualizes care. This is optimized by systematic assessment of social drivers of health with coordinated follow-up and re-evaluation through recurrent discussions. The IP-DEMP model is flexible, offering individual in-person and telehealth modes of curriculum delivery; the latter has been demonstrated as a safe effective mode of GDM treatment.^24^ Colocation bolsters seamless integrated care delivery and facilitates direct and ongoing communication between IP-DEMP team members and prenatal providers.

Consistently high participant engagement underscores the strengths of the IP-DEMP. In 2022, the IP-DEMP referral office that serves patients primarily with Medicaid coverage (>90%) had a missed appointment rate of 22% (pooled mean, range 9.1–34.1%; Table 3). This stands in contrast to the generally low appointment adherence among Medicaid-insured patients; a 2018–2019 study indicated that a proportion of 30.5% (95% confidence interval=30.2–30.9%) of Medicaid-insured patients in a northeastern urban health system missed their primary care appointments.^25^

Although DM educators may not typically schedule their own appointments in health care systems, the high level of patient engagement associated with the IP-DEMP workflow lends support for this approach, likely bolstered through consistency and continuity through ongoing management from the same team member. Initial outreach to explain new diagnosis and schedule initial appointment occurs within 2 business days after receiving referral, with the initial appointment occurring within 1 week of referral. Follow-up appointments are scheduled every 1–2 weeks.

DM-related postpartum care, such as oral glucose tolerance testing, ongoing glucose monitoring, and preventive DM intervention discussions, is crucial to reducing long-term adverse effects of perinatal DM.^26^ About 35–50% of people with GDM have an increased risk of recurrence^27^; GDM engenders a 7-to-10-fold increased risk of T2DM development.^5,26,27^ Nevertheless, postpartum care engagement is low^3,26^: in a cohort study, just 50.9% and 36.2% of postpartum patients with GDM received primary care and DM-related care follow-up, respectively—lower rates than for patients with pre-existing T2DM.^26^

In contrast, the IP-DEMP maintained high postpartum follow-up adherence rates in 2022, averaging at 72.5% and 72.0% for patients insured by Medicaid and commercial plans, respectively (Table 3). This is most likely due to education and coordination of postpartum care before delivery.

## Discussion

### Contribution to intervention models

Initial patient responses to the IP-DEMP model align with prior evidence that DSMES programs may help to improve overall quality of life and self-efficacy more effectively than routine obstetrical care.^8,27^ Interventions including dietary counseling, glucose monitoring, gestational weight gain monitoring, and/or insulin therapy have been evaluated in randomized controlled trials among pregnant persons, yielding improved health outcomes and reduced adverse events.^3,8,27^ The IP-DEMP offers promising early evidence that this approach can be successfully generalized. Given the greater burden of DM among historically marginalized groups, improved services to birthing individuals with DM may offer a strategy to improve maternal health inequities.

## Implications for Practice and Policy

### Future directions

This study describes the IP-DEMP model and its process metrics. Program evaluation is needed to establish quality improvements associated with program implementation. Analysis of characteristics of participants who engage with the program, including insurance type, and empirical evaluation of clinical outcomes is ongoing.

### Sustainability

Medicaid significantly impacts access to DM education. Compared with privately insured counterparts, Medicaid recipients are more likely to be people of color and enter pregnancy with pre-existing DM or obesity.^28^ Yet, Medicaid does not cover DSMES services in 17 states,^29^ including in the District of Columbia where the intervention was implemented. Nonetheless, the IP-DEMP was able to utilize nutrition education codes to bill for DM education visits, and the program coordinator is working with the Association of Diabetes Care and Education Specialists (ADCES) and other local organizations to advocate for DSMES coverage for Medicaid recipients.

Considering the benefits of DSMES, the aforementioned states can be expected to improve maternal, fetal, and infant health by securing DSMES coverage through legislation, administrative code, or budget documents; integrating DSMES as a covered service in their Medicaid State Plan; and delivering DSMES through Medicaid-managed care.^29^

### Lessons learned

Several key lessons were learned during development and implementation. It is imperative to have a formal streamlined referral process, with clear diagnostic criteria before launching a similar program. In addition, national accreditation through the ADA or ADCES allows for increased reimbursement in addition to access to a wide array of patient education materials. As part of this process, consolidation of an integrated DM program under one entity that serves the entire population is beneficial to reduce confusion and allow for oversight.

For example, instead of accessing individual prenatal care providers, aligning IP-DEMP under MFM allows access to a broad swath of patients. Finally, involving key stakeholders before program implementation allows all involved parties (clinicians, educators, social workers, case managers, nurses, and mental health care providers) to identify necessary aspects of patient care and allow for a unified care model.

## Conclusions

DM care interventions play a vital role in helping patients manage their health.^1,8,9^ IP-DEMP offers colocated office visits during prenatal and postpartum care and telemedicine appointments to engage participants in ongoing clinical management throughout the perinatal period. High levels of engagement are achieved, likely through direct outreach from the team, continuity, and colocation with the prenatal team; coordination before delivery likely promotes postpartum engagement. Integrating DM care within the obstetrical setting offers a promising approach that may contribute to efforts to achieve equity in outcomes for the birthing individual, fetus, and newborn.

## Abbreviations Used

ADA: American Diabetes Association
ADCES: Association of Diabetes Care and Education Specialists
CDCES: certified diabetes care and education specialist
DM: diabetes mellitus
DSMES: diabetes self-management education and support
EMR: electronic medical record
GDM: gestational diabetes mellitus
IP-DEMP: integrated perinatal diabetes education and management program
MFM: maternal–fetal medicine
